# Effect of Time of Day on Sustained Postexercise Vasodilation Following Small Muscle-Mass Exercise in Humans

**DOI:** 10.3389/fphys.2019.00762

**Published:** 2019-06-25

**Authors:** Leandro C. Brito, Matthew R. Ely, Dylan C. Sieck, Joshua E. Mangum, Emily A. Larson, Christopher T. Minson, Cláudia L. M. Forjaz, John R. Halliwill

**Affiliations:** ^1^Exercise Hemodynamic Laboratory, School of Physical Education and Sport, University of São Paulo, São Paulo, Brazil; ^2^Department of Human Physiology, University of Oregon, Eugene, OR, United States

**Keywords:** regional blood flow, aerobic exercise, circadian rhythm, diurnal variation, hemodynamic

## Abstract

**Introduction:**

Previous studies observed diurnal variation in hemodynamic responses during recovery from whole-body exercise, with vasodilation appearing greater after evening versus morning sessions. It is unclear what mechanism(s) underlie this response. Since small muscle-mass exercise can isolate peripheral effects related to postexercise vasodilation, it may provide insight into possible mechanisms behind this diurnal variation.

**Methods:**

The study was conducted in ten healthy (5F, 5M) young individuals, following single-leg dynamic knee-extension exercise performed in the Morning (7:30–11:30 am) or the Evening (5–9 pm) on two different days, in random order. Arterial pressure (automated auscultation) and leg blood flow (femoral artery Doppler ultrasound) were measured pre-exercise and during 120 min postexercise. Net effect for each session was calculated as percent change in blood flow (or vascular conductance) between the Active Leg and the Inactive Leg.

**Results:**

Following Morning exercise, blood flow was 34.9 ± 8.9% higher in the Active Leg versus the Inactive Leg (*p* < 0.05) across recovery. Following Evening exercise, blood flow was 35.0 ± 8.8% higher in the Active Leg versus the Inactive Leg (*p* < 0.05). Likewise, vascular conductance was higher in the Active Leg versus the Inactive Leg (Morning: +35.1 ± 9.0%, *p* < 0.05; Evening: +33.2 ± 8.2%, *p* < 0.05). Morning and Evening blood flow (*p* = 0.66) and vascular conductance (*p* = 0.64) did not differ.

**Conclusion:**

These data suggest previous studies which identified diurnal variations in postexercise vasodilation responses are likely reflecting central rather than peripheral modulation of cardiovascular responses.

## Introduction

Blood flow and vascular conductance remain elevated up to 2 h after an acute session of aerobic exercise (Halliwill et al., 2013; Romero et al., 2017). This sustained postexercise vasodilation of skeletal muscle vascular beds has been observed after both whole-body exercise (e.g., cycling or running) (Lockwood et al., 2005a,b; McCord and Halliwill, 2006; de Brito et al., 2015; Ely et al., 2017) and small muscle-mass exercise (e.g., single-leg dynamic knee-extension) (Barrett-O’Keefe et al., 2013; Romero et al., 2015). Depending on the form of exercise, the underlying mechanisms of sustained postexercise vasodilation can differ. For example, after small muscle-mass exercise histamine is produced and released in the active skeletal muscles and induces vasodilation through interaction with histamine H_1_- and H_2_-receptors (McCord and Halliwill, 2006; Ely et al., 2017). Whereas in whole-body exercise, postexercise reductions in sympathetic nerve activity further contributes to this histaminergic vasodilation (Halliwill et al., 1996a; Forjaz et al., 1999).

As most aspects of cardiovascular regulation demonstrate a circadian or diurnal pattern (Portaluppi and Hermida, 2007), it is possible the mechanisms that drive sustained postexercise hypotension are impacted by time of day. Circadian variation promotes lower levels of sympathetic activity (Grassi et al., 2010) and higher concentrations of vasodilating factors such as histamine (Rehn et al., 1987; Asano et al., 1995) in the early evening, which may favor greater vasodilation when exercise is performed at this time of day. Additionally, a greater reactive hyperemia response and fall in total vascular resistance was observed after whole-body exercise in the evening than in the morning (de Brito et al., 2015). In contrast, flow-mediated dilation was blunted following afternoon exercise but unchanged following morning exercise (Jones et al., 2010). These initial observations are consistent with diurnal variation in exercise responses, but difficult to interpret as vascular responses to whole-body exercise are regulated by multiple mechanisms, such as baroreflex resetting (Halliwill et al., 1996b), sympathoinhibition (Halliwill et al., 1996a; Forjaz et al., 1999), and histamine dependent-vasodilation (Lockwood et al., 2005b; McCord and Halliwill, 2006; Romero et al., 2015; Ely et al., 2017). Thus, isolating one of these mechanisms would help to further elucidate which avenues diurnal variation may act through to influence the vascular response to exercise. This information may contribute to understand the vascular response to a stimulus at different times of day, which may guide therapy strategies or administration of medications. Along these lines, we have previously shown that a standardized small muscle-mass exercise protocol using single-leg dynamic knee-extension is an effective model to test for changes in local blood flow and vascular conductance in response to exercise (Barrett-O’Keefe et al., 2013; Romero et al., 2015), and might be sensitive to detect diurnal variation in the histaminergic component of sustained postexercise vasodilation.

Therefore, the aim of this study was to compare leg blood flow and vascular conductance after a 1 h session of single-leg dynamic knee-extension exercise, performed in the morning and in the evening. Our hypothesis was that leg blood flow and vascular conductance would be higher after exercise in both the morning and the evening, unchanged in the control non-active leg, and that this rise in blood flow and vascular conductance would be greater after evening, compared to morning exercise.

## Materials and Methods

### Subjects

Ten young healthy non-smoking subjects (5 females, 5 males) volunteered for the current study. Subject’s health status was confirmed through a standard health history questionnaire. All subjects were deemed sedentary or recreationally active based on their exercise habits in the previous 12 months as assessed by two self-reported questionnaires (Baecke et al., 1982; Kohl et al., 1988). No subjects had extreme morningness (score > 69) or eveningness (score < 31) chronotypes as assessed by a self-reported questionnaire (Horne and Ostberg, 1976). Thus, we minimized the potential confounding influence of extreme “night owls and early birds” on the study outcome. Females were not pregnant as confirmed by a negative pregnancy test before every study visit. No subjects were taking over-the-counter or prescription medications at the time of the study, with the exception of oral contraceptives. Female subjects were investigated during the early follicular phase of their menstrual cycle. This study was approved by the Institutional Review Board of the University of Oregon (Protocol #02172011.029). All subjects signed the informed consent prior to participation and the study was conducted in accordance with the latest revision of the Declaration of Helsinki, and was not registered in a database.

### Screening Visit

During this visit, subject’s physical characteristics were obtained (height, weight, body mass index) and they were familiarized with the exercise model and hemodynamic measurements. Arterial pressure was assessed in triplicate using an automated auscultatory sphygmomanometer (Tango+; SunTech Medical, Raleigh, NC, United States) after 5 min of supine rest. Leg blood flow was assessed by duplex ultrasonography using a linear-array ultrasound transducer (L9-3 probe, Philips iE33, Andover, MA, United States) to identify possible anatomical difficulties and to familiarize subjects with the procedure.

All subjects underwent a peak single-leg dynamic knee-extension exercise test to volitional fatigue to determine the work rate for the study days. Exercise was performed using a custom-built knee extension ergometer based on a computer-controlled step-motor that generated resistance against the subject’s lower leg as previously described (Barrett-O’Keefe et al., 2013; Romero et al., 2015). Subjects were seated in an upright position and they were instructed to perform knee-extensions with their right leg over a 45° range of motion, starting with the leg hanging at ∼90° of flexion, and maintain a cadence of 45 extensions per minute, following audio and visual feedback. Workload was increased 3 W every minute until the subject could not keep up the required cadence or range of motion. This exercise model produced volitional fatigue in 6.4 ± 2.0 min (range 5–11 min).

### Experimental Protocol

Subjects reported to the laboratory on two study days, one in the Morning (7:30–11:30 am.) and one in the Evening (5–9 pm.). Conditions were randomized and separated by 3 to 7 days. Subjects were instructed to keep similar routines and to abstain from alcohol, caffeine, and exercise for 24 h before each experiment. Subjects were also required to arrive at the laboratory having fasted for at least the previous 4 h. Room temperature was controlled in all experimental sessions (∼20°C). The testing area had no windows; therefore, luminosity was kept the same for all experimental sessions.

After arriving at the laboratory, subjects rested in the supine position for 10 min before the start of measurements. Leg blood flow (in each leg) and arterial pressure were measured twice 30 min before exercise, and every 20 min during the 120 min after exercise. The exercise protocol consisted of performing single-leg dynamic knee-extension exercise with the right leg for 60 min at 60% of peak power at a cadence of 45 knee-extensions min^−1^. Power was ramped at the onset of exercise to 60% peak power over the first 5 min. Power output was recorded continuously throughout the 60 min dynamic knee-extension exercise.

### Measurements

All hemodynamic measurements were performed with subjects remaining quiet and relaxed in the supine position.

#### Heart Rate and Arterial Pressure

Heart rate was monitored using a three-lead electrocardiograph and arterial pressure was assessed in the right arm employing an automated sphygmomanometer (Tango+; SunTech Medical, Raleigh, NC, United States). Heart rate and arterial pressure were recorded in triplicate before every leg blood flow measurement. Mean arterial pressure was calculated as (systolic – diastolic)/3 + diastolic pressure.

#### Leg Blood Flow and Vascular Conductance

Pressure cuffs (Hokanson E20 Rapid Cuff Inflator; D. E. Hokanson, Inc., Bellevue, WA, United States) around the ankle of each leg were inflated to 250 mmHg to occlude the foot circulation 1 min prior to and during measurements, to avoid interference from blood flowing in arteriovenous anastomoses of the feet. Femoral artery blood flow velocity was determined 2–3 cm proximal to the common femoral artery bifurcation via duplex ultrasonography using a linear-array ultrasound transducer (L9-3 probe, Philips iE33, Andover, MA, United States) with an insonation angle of 60°. Custom ultrasound software was used to capture the forward and reverse Doppler-shifted signals from the ultrasound system. Recordings were subsequently analyzed with an intensity-weighted algorithm to determine mean blood velocity following standard methods for quantification (Buck et al., 2014). Velocity measurements were assessed at an average depth of 1.91 ± 0.48 cm and were corrected for beam-width of 3.13 ± 0.24 mm, which resulted in an average correction factor of 0.790 ± 0.011 (Buck et al., 2014). Average femoral artery diameter was determined by automated edge-detection software (Vascular Research Tools 5 – Version 6.1.1. Medical Imaging Application LLC, Coralville, IA, United States). Leg blood flow (ml min^−1^) was calculated as the product of femoral artery cross-sectional area mean femoral blood velocity. Leg vascular conductance (ml min^−1^ mmHg^−1^) was calculated by dividing leg blood flow by mean arterial pressure.

### Statistical Analysis

Subjects were not separated by sex as the small cohort had insufficient power for testing sex differences and previous studies found no sex difference in postexercise leg blood flow (Barrett-O’Keefe et al., 2013; Romero et al., 2015; Ely et al., 2017). The normal distribution of data was confirmed by Shapiro–Wilk test. The average of measurements taken prior to exercise was defined as baseline for heart rate, arterial pressures, leg blood flow, and leg vascular conductance. We used a two-way (for heart rate and arterial pressures) or three-way (for leg blood flow and leg vascular conductance) repeated-measures ANOVA with *a priori* contrasts to analyze differences in session (Morning vs. Evening), condition (Active Leg vs. Inactive Leg), and time (Pre vs. Postexercise). In addition, we leveraged the concurrent responses in the Active Leg versus the Inactive Leg by calculating a relative net effect during recovery from exercise as (Active_post_-Active_pre_)/Active_pre_ – (Inactive_post_-Inactive_pre_)/Inactive_pre_, which is the percent change in blood flow (or vascular conductance) between the Active Leg and the Inactive Leg referenced to the pre-exercise values for both legs. We modeled the postexercise leg blood flow, leg vascular conductance, and net effects during recovery from exercise across session (Morning and Evening) and time using a stepwise regression which was run with SAS Proc GLMSELECT (SAS version 9.2; SAS Institute Inc., Cary, NC, United States). This approach allowed examination of both linear and quadratic relationships across time, and tested whether or not the relationships differed between the sessions, as demonstrated previously (Romero et al., 2015; Ely et al., 2017). Independent variables remained in the model if a minimal *P-*value threshold was met (*P* < 0.15). Significance was set at *P* < 0.05. Data are reported as means ± standard error (SE), except for data characterizing the subjects which are presented as means ± standard deviation (SD).

## Results

Subject’s characteristics and data obtained during the screening visit are shown in Table 1. Subjects matched a physical activity level corresponding to recreationally active. No subject was classified as an extreme chronotype, as 1 was classified as E-type (favor eveningness), 4 as M-type (favor morningness), and 5 were intermediate (not favoring either chronotype).

**Table 1:** Characteristics of the subjects.

N	10(5M/5F)
Age (years)	25 5
Height (m)	1.73 0.11
Weight (kg)	67.7 13.4
Body mass index (kg/m^2^)	22.6 2.5
Systolic arterial pressure (mmHg)	107 8
Diastolic arterial pressure (mmHg)	72 5
Baecke sport index (quintile score)	3.0 1.0
Physical activity index (MET h^−1^ week^−1^)	33.6 23.3
Chronotype (score)	54.8 9.9
Peak power output (W)	24.6 7.9

*Values are mean ± SD.*

### Pre-exercise

Pre-exercise mean arterial pressure and heart rate were similar between morning and evening experimental sessions, as shown in Table 2.

**Table 2:** Central hemodynamics pre- and post-exercise performed in the morning and the evening.

Time point	Heart rate (bpm)		Mean arterial pressure (mmHg)	
	Morning	Evening	Morning	Evening
Pre-exercise	55.8 ± 3.3	57.9 ± 3.3	80.4 ± 2.2	80.7 ± 2.0
Time postexercise				
20 min	56.4 ± 3.2	59.1 ± 3.2	80.3 ± 1.3	81.2 ± 2.6
40 min	55.2 ± 3.1	56.1 ± 3.2	79.9 ± 1.1	80.8 ± 2.3
60 min	53.7 ± 3.0	56.3 ± 3.3	79.0 ± 1.3	82.0 ± 2.6
80 min	53.9 ± 2.7	55.3 ± 3.7^∗^	80.6 ± 1.9	83.6 ± 2.6
100 min	53.5 ± 2.6	54.4 ± 3.2^∗^	80.5 ± 2.2	83.2 ± 2.9
120 min	54.1 ± 3.0	54.9 ± 3.1^∗^	83.0 ± 2.2	85.9 ± 2.7^∗^

*Values are means ± SE; n = 10; ^∗^P < 0.05 vs. pre-exercise across sessions (there were no differences between Morning and Evening sessions).*

Prior to exercise, leg blood flow did not differ (*P* = 0.68) between the Active Leg (202 ± 15 ml min^−1^) and the Inactive Leg (210 ± 15 ml min^−1^) or differ (*P* = 0.25) between Morning (194 ± 14 ml min^−1^) and Evening (218 ± 18 ml min^−1^) sessions. Likewise, leg vascular conductance did not differ prior to exercise (*P* = 0.61) between the Active Leg (2.50 ± 0.17 ml min^−1^ mmHg^−1^) and the Inactive Leg (2.62 ± 0.17 ml min^−1^ mmHg^−1^) or differ (*P* = 0.24) between Morning (2.41 ± 0.17 ml min^−1^ mmHg^−1^) and Evening (2.71 ± 0.20 ml min^−1^ mmHg^−1^) sessions.

### Exercise Responses

Subjects were supervised during exercise to ensure they kept similar power output (60% of peak power) during steady-state dynamic knee-extension exercise for both visits. While exercising, heart rate (Morning: 83.4 ± 4.6 vs. Evening: 84.3 ± 4.5 beats min^−1^, *P* = 0.70) and mean arterial pressure (Morning: 98.4 ± 2.9 vs. Evening: 102.4 ± 2.3 mmHg, *P* = 0.26) were not different between Morning and Evening sessions.

### Central Hemodynamics

Table 2 shows heart rate and mean arterial pressure pre-exercise and during recovery from exercise. While both variables changed across the 2-h time-course of recovery, there were no differences between Morning and Evening sessions for mean arterial pressure (*P* = 0.55) or heart rate (*P* = 0.61). Heart rate decreased from 80 to 120 min postexercise compared to pre-exercise (*P* < 0.05), while mean arterial pressure was higher at 120 min postexercise compared to pre-exercise (*P* < 0.05).

### Leg Blood Flow and Vascular Conductance

**FIGURE 1:**
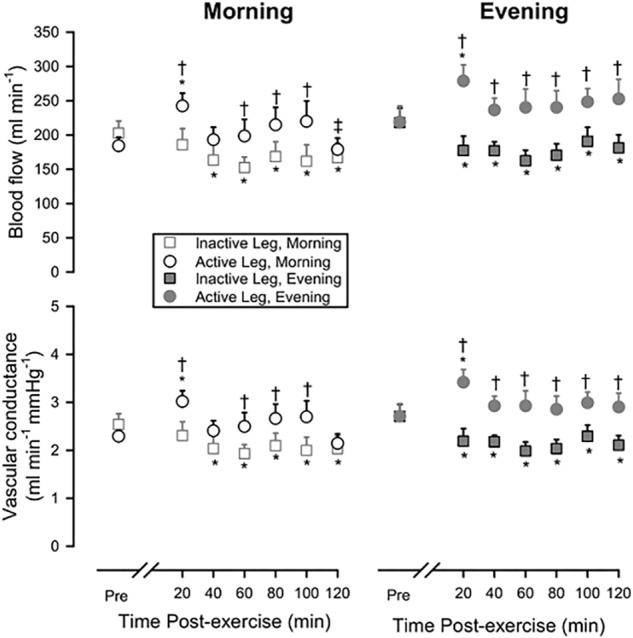
Leg blood flow (upper panels) and leg vascular conductance (lower panels) pre-exercise and during 120 min of recovery from exercise in Morning (left panels) or Evening (right panels) sessions, showing both the response in the Active Leg and the Inactive Leg. Open squares: Inactive Leg during Morning session; Open circles: Active Leg during Morning session; Filled squares: Inactive Leg during Evening session; Filled circles: Active Leg during Evening session. Prior to exercise, blood flow and vascular conductance did not differ between Active Leg and Inactive Leg or between Morning and Evening sessions. ^∗^*P* < 0.05 vs. pre-exercise; ^†^*P* < 0.05 Active Leg vs. Inactive Leg; ^‡^*P* < 0.05 Morning vs. Evening. Means ± SE; *n* = 10.

Figure 1 shows leg blood flow and leg vascular conductance pre-exercise and during recovery from exercise, comparing the Active Leg to the Inactive Leg during recovery from exercise in the Morning and Evening. During both sessions, blood flow and vascular conductance were reduced (*P* < 0.05) following exercise in the Inactive Leg across much of the recovery period, whereas blood flow and vascular conductance were increased (*P* < 0.05) in the Active Leg early after exercise.

Following Morning exercise, blood flow was 34.9 ± 8.9% higher in the Active Leg compared to the Inactive Leg (*P* < 0.05) across the 2-h time-course of recovery. Following Evening exercise, blood flow was 35.0 ± 8.8% higher in the Active Leg compared to the Inactive Leg (*P* < 0.05) across the 2-h time-course of recovery. Likewise, vascular conductance was higher following exercise in the Active Leg compared to the Inactive Leg (Morning: +35.1 ± 9.0%, *P* < 0.05; Evening:+33.2 ± 8.2%, *P* < 0.05).

**FIGURE 2:**
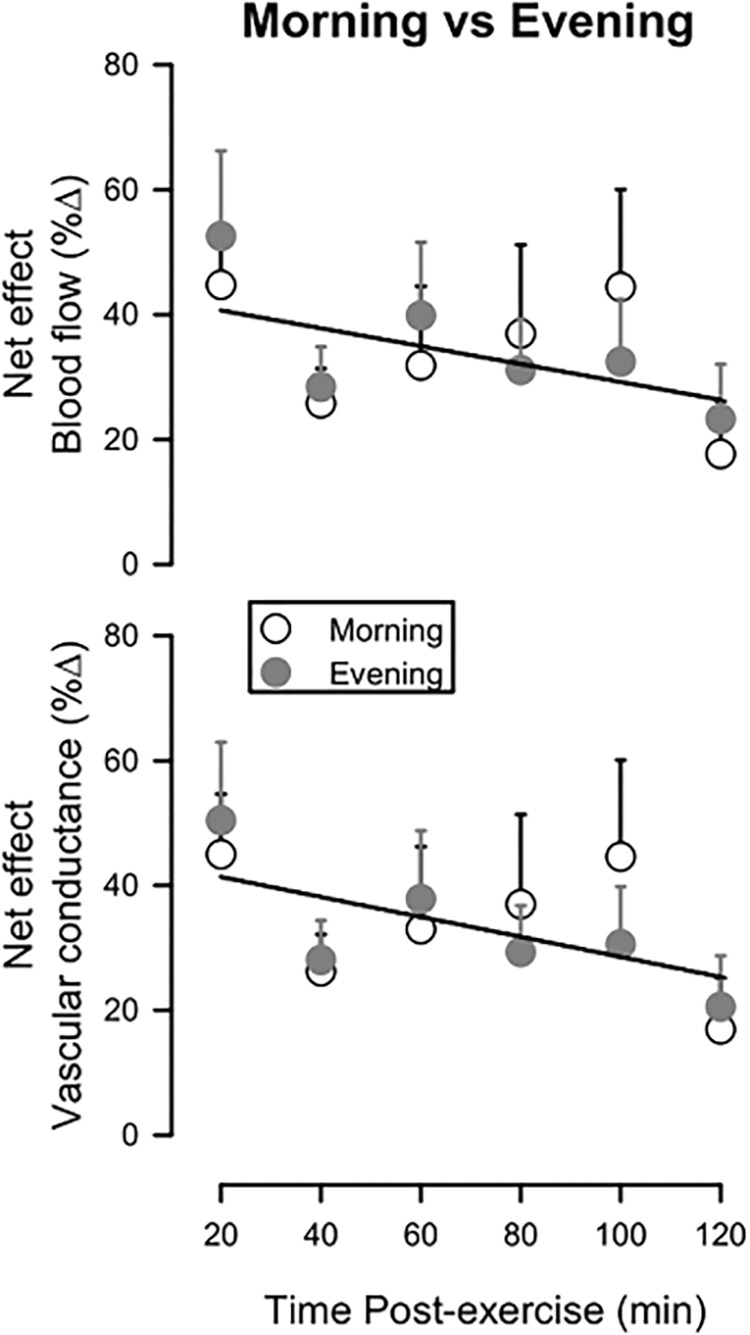
Net effect for leg blood flow (upper panel) and leg vascular conductance (lower panel) during 120 min of recovery from exercise, expressed as a percent change between the Active Leg and the Inactive Leg, referenced to pre-exercise values. Open circles: Morning session; Filled circles: Evening session. Solitary regression lines for the net effect for blood flow and vascular conductance indicate the absence of differences between Evening and Morning sessions. Means ± SE; *n* = 10.

Figure 2 depicts how these net effects (the percent change in blood flow or vascular conductance between the Active Leg and the Inactive Leg referenced to the pre-exercise values for both legs) vary across the 2-h time-course of recovery, as well as how they compare between the Morning and Evening sessions. For both blood flow and vascular conductance, net effects were highest early after exercise and showed a trend to decrease over time (*P* = 0.10 for blood flow; *P* = 0.08 for vascular conductance). The net effects did not differ between Morning and Evening sessions for either blood flow (*P* = 0.66) or vascular conductance (*P* = 0.64).

## Discussion

The main finding of this study was that postexercise vasodilation after a single bout of dynamic knee-extension exercise is unaltered whether the exercise session takes place in the morning or evening. This is in contrast to our hypothesis that postexercise vasodilation would be greater after evening than morning exercise, and also differs from what has been observed in studies that measured vascular responses following whole-body exercise at different times of day (Jones et al., 2010; de Brito et al., 2015). However, we note further observations from our data, which need to also be considered. First, the consistent and robust vasodilation in the previously exercised leg (Barrett-O’Keefe et al., 2013; Romero et al., 2015) was superimposed on postexercise vasoconstriction (evident in the inactive leg). Second, a somewhat higher blood flow and vascular conductance pre-exercise in the evening, albeit not statistically significant, can give the false impression of greater responses of these variables postexercise in the evening compared to morning. This illusion is mitigated by using the net effect analysis (Figure 2). Overall, our results contribute to the concept that aerobic exercise promotes local vascular changes, which are likely beneficial, after both morning and evening exercise.

Concerning the lower vasodilation observed in this study compared to previous studies from our group (Barrett-O’Keefe et al., 2013; Romero et al., 2015), the increase of the mean arterial pressure and heart rate postexercise at both times of day (Table 2), and the vasoconstriction in the Inactive Leg (Figure 1) suggested an increase of sympathetic activity. In our prior work, this model of exercise did not affect baroreflex sensitivity, so we do not think there is a resetting of the baroreflex or reductions in peripheral sympathetic activity as seen with whole-body exercise (Buck et al., 2015). Thus, we suspect that subjects may have become physically uncomfortable or restless over the time course of the study, resulting in a gradual rise in sympathetic nerve activity and generalized vasoconstriction. We are unable to determine what aspect of our study design might have contributed to this.

### Mechanisms of Sustained Postexercise Vasodilation

It is well established that a single session of whole-body aerobic exercise can promote postexercise vasodilation which results in elevated blood flow and is associated with postexercise hypotension (Halliwill et al., 2013; Romero et al., 2017). Postexercise vasodilation occurs in both the vascular beds of previously active skeletal muscles (i.e., leg or calf blood flow) and inactive skeletal muscles (i.e., forearm blood flow) following activities such as cycling (Halliwill et al., 2000; McCord and Halliwill, 2006). However, postexercise vasodilation is not observed in other vascular regions, such as the splanchnic, renal, and skin vascular beds (Pricher et al., 2004; Buck et al., 2015).

Curiously, the inactive leg presented vasoconstriction in the present study, which was not observed in the previous studies using this model of exercise (Barrett-O’Keefe et al., 2013; Romero et al., 2015). This vasoconstriction might be reflected in the increased mean arterial pressure after exercise at both times of day, but only after 120 min of recovery (Table 2). Thus, it is not an effect of exercise, but an effect of the time since it occurred at the end if this period.

These unexpected responses suggest that there could be an increase of sympathetic activity during the recovery period in the present study. Since this model of exercise does not promote changes in baroreflex resetting (Buck et al., 2015), it is possible that subjects were becoming physically uncomfortable or feeling restless, resulting in a sympathoexcitatory response toward the end of the long recovery period, which might be explained by the increase in the mean arterial pressure at 120 min after exercise. As we have not always observed this pattern, it seems unlikely that it represents an exercise-generated central regulatory response, but something that was consistent between the morning and the evening sessions.

Both central and peripheral mechanisms are involved in skeletal muscle vasodilation (Halliwill et al., 2013; Romero et al., 2017), including resetting of the arterial baroreflex operating point (Halliwill et al., 1996a), decreasing peripheral sympathetic vasoconstrictor nerve activity (Halliwill et al., 1996a; Forjaz et al., 1999), possible attenuation of presynaptic release of norepinephrine [as the sensitivity of α-adrenergic receptors are preserved, yet vascular transduction is blunted (Jones et al., 2008)], and release of local vasodilators.

Prior studies on the contribution of local vasodilators found, while nitric oxide (Halliwill et al., 2000) and prostaglandins (Lockwood et al., 2005a) are not necessary to evoke sustained postexercise vasodilation, histamine H_1_ and H_2_ receptors (Lockwood et al., 2005a,b; McCord and Halliwill, 2006; Ely et al., 2017) play an important role after moderate intensity aerobic exercise. Histamine is quickly metabolized, and is generally not elevated in either plasma or whole blood following exercise (Lockwood et al., 2005b; McCord and Halliwill, 2006), such that its effects on H_1_ and H_2_ receptor are localized during recovery from exercise to the previously exercised muscle. Within exercising muscle, histamine appears to be locally released by mast cells and as well as generated by histidine decarboxylase (Romero et al., 2017). It has been suggested that reactive oxygen and nitrogen species produced in skeletal muscle during contractions (Powers et al., 2011) due to an imbalance between pro-oxidants and antioxidant defense mechanisms altering redox control (Jones, 2008), as documented in response to whole-body exercise (Dillard et al., 1978) and single-leg dynamic knee-extension exercise (Bailey et al., 2007), might contribute to postexercise vasodilation (New et al., 2013). However, antioxidants do not alter postexercise vasodilation in humans (Romero et al., 2015).

Thus, the current understanding is that locally released histamine is responsible for sustained vasodilation in previously exercised muscles, while baroreflex resetting and sympathoinhibition contribute to vasodilation in both the active and inactive muscles. Whole-body exercise results in a vasodilation postexercise in active and inactive muscle groups, involving vasodilatory substances, such as histamine, but also with a decrease in peripheral sympathetic activity promoted by resetting of baroreflex control. Single-leg exercise promotes vasodilation only in active muscle groups involved in the exercise and this seems to be mainly due to the histaminergic receptors activity, as seen in the previous studies (Barrett-O’Keefe et al., 2013; Romero et al., 2015). This model of exercise eliminates the contribution of baroreflex resetting (Buck et al., 2015), vasodilation is not evident in the inactive muscle vascular beds, which sometimes shows evidence of vasoconstriction (as in the present study).

### Diurnal Differences With Whole-Body Exercise

In contrast to the present study, prior research using whole-body exercise found a greater reactive hyperemia response after evening than morning exercise, as well as a greater postexercise fall in total vascular resistance (Jones et al., 2008; de Brito et al., 2015). The coupling of this prior research with the observations of the present study suggests that while diurnal variation in exercise responses can occur, they are likely independent of histaminergic signaling that generates sustained postexercise vasodilation. The vasodilation promoted by this model of aerobic exercise is abolished if histaminergic receptors are blocked (Barrett-O’Keefe et al., 2013). Then, differences between morning and evening vasodilation observed in previous studies may also reflect central modulation of exercise responses rather than peripheral signaling associated with the exercised muscle, such as is possible to observe after whole-body exercise (Halliwill et al., 1996a). Along this line, Buck et al. (2015) reported that this single-leg small muscle-mass aerobic exercise does not promote central changes in baroreflex that reflects in decreasing peripheral sympathetic activity to the active muscle.

## Conclusion

An acute bout of single-leg dynamic knee-extension exercise in healthy young adults promotes sustained postexercise vasodilation in the morning and in the evening, and the response is not different between morning and evening exercise. This suggests that previously identified diurnal variations in postexercise vascular responses are more likely due to central than peripheral modulation.

## Data Availability

The raw data supporting the conclusions of this manuscript will be made available by the authors, without undue reservation, to any qualified researcher.

## Ethics Statement

This study was approved by the Institutional Review Board of the University of Oregon (Protocol #02172011.029). All subjects signed the informed consent prior to participation and the study was conducted in accordance with the latest revision of the Declaration of Helsinki, and was not registered in a database.

## Author Contributions

LB, CF, CM, and JH were responsible for the conception and design of the study. LB, ME, DS, JM, and EL were responsible for the collection and analysis of the data. LB, CF, and JH were responsible for interpretation of the data. LB, ME, DS, JM, and EL drafted the manuscript. CM, CF, and JH critically revised the same. The final version of the manuscript was approved by all the authors. All researchers designed as author qualify for authorship, and all those who qualify for authorship are listed.

## Conflict of Interest Statement

The authors declare that the research was conducted in the absence of any commercial or financial relationships that could be construed as a potential conflict of interest.
